# Supplementary material for: Resolving the full spectrum of human genome variation using Linked-Reads
Source: Genome Res. 2019 Apr;29(4):635–45. doi: 10.1101/gr.234443.118 (PMC6442396; doi:10.1101/gr.234443.118)
Supplement: Supplemental Material [file supp_gr.234443.118_Supplemental_longranger-2.2.zip › longranger-2.2.tar.bz2!/lr2.2-public/lib/go/src/code.google.com/vitess/docs/user-guide/vitess-sequences.html]

Vitess / Vitess Sequences


Toggle navigation

- Overview
- Guides
- Reference
- Blog
- GitHub

- #### Overview

  - What is Vitess
  - Scaling MySQL with Vitess
  - Key Concepts
- #### Getting Started

  - Run Vitess on Kubernetes
    - Custom Docker Build
  - Run Vitess Locally
- #### User Guide

  - Introduction- Client Libraries- Backing Up Data- Reparenting
        - Schema Management
        - VSchema Guide
        - Schema Swap (Tutorial)
        - Sharding
          - Horizontal Sharding (Tutorial, automated)
          - Horizontal Sharding (Tutorial, manual)
          - Sharding in Kubernetes (Tutorial, automated)
          - Sharding in Kubernetes (Tutorial, manual)
        - Vitess Sequences
        - MySQL Server Protocol
        - Vitess and Replication
        - Update Stream
        - Row Based Replication
        - Topology Service
        - Transport Security Model
        - Launching
          - Scalability Philosophy
          - Production Planning
          - Server Configuration
          - 2PC Guide
          - Troubleshooting
        - Upgrading
- #### Reference Guides

  - Vitess API- vtctl Commands
- #### Other Resources

  - Presentations- Blog- Roadmap
- #### Contributing

  - Contributing to Vitess- GitHub Workflow- Code Reviews
- GitHub

# Vitess Sequences

- #### Overview

  - What is Vitess
  - Scaling MySQL with Vitess
  - Key Concepts
- #### Getting Started

  - Run Vitess on Kubernetes
    - Custom Docker Build
  - Run Vitess Locally
- #### User Guide

  - Introduction- Client Libraries- Backing Up Data- Reparenting
        - Schema Management
        - VSchema Guide
        - Schema Swap (Tutorial)
        - Sharding
          - Horizontal Sharding (Tutorial, automated)
          - Horizontal Sharding (Tutorial, manual)
          - Sharding in Kubernetes (Tutorial, automated)
          - Sharding in Kubernetes (Tutorial, manual)
        - Vitess Sequences
        - MySQL Server Protocol
        - Vitess and Replication
        - Update Stream
        - Row Based Replication
        - Topology Service
        - Transport Security Model
        - Launching
          - Scalability Philosophy
          - Production Planning
          - Server Configuration
          - 2PC Guide
          - Troubleshooting
        - Upgrading
- #### Reference Guides

  - Vitess API- vtctl Commands
- #### Other Resources

  - Presentations- Blog- Roadmap
- #### Contributing

  - Contributing to Vitess- GitHub Workflow- Code Reviews

# Vitess Sequences

This document describes the Vitess Sequences feature, and how to use it.

## Motivation

MySQL provides the `auto-increment` feature to assign monotonically incrementing
IDs to a column in a table. However, when a table is sharded across multiple
instances, maintaining the same feature is a lot more tricky.

Vitess Sequences fill that gap:

- Inspired from the usual SQL sequences (implemented in different ways by
  Oracle, SQL Server and PostgreSQL).
- Very high throughput for ID creation, using a configurable in-memory block allocation.
- Transparent use, similar to MySQL auto-increment: when the field is omitted in
  an `insert` statement, the next sequence value is used.

## When *not* to Use Auto-Increment

Before we go any further, an auto-increment column has limitations and
drawbacks. let's explore this topic a bit here.

### Security Considerations

Using auto-increment can leak confidential information about a service. Let's
take the example of a web site that store user information, and assign user IDs
to its users as they sign in. The user ID is then passed in a cookie for all
subsequent requests.

The client then knows their own user ID. It is now possible to:

- Try other user IDs and expose potential system vulnerabilities.
- Get an approximate number of users of the system (using the user ID).
- Get an approximate number of sign-ins during a week (creating two accounts a
  week apart, and diffing the two IDs).

Auto-incrementing IDs should be reserved for either internal applications, or
exposed to the clients only when safe.

### Alternatives

Alternative to auto-incrementing IDs are:

- use a 64 bits random generator number. Try to insert a new row with that
  ID. If taken (because the statement returns an integrity error), try another
  ID.
- use a UUID scheme, and generate trully unique IDs.

Now that this is out of the way, let's get to MySQL auto-increment.

## MySQL Auto-increment Feature

Let's start by looking at the MySQL auto-increment feature:

- A row that has no value for the auto-increment value will be given the next ID.
- The current value is stored in the table metadata.
- Values may be ‘burned’ (by rolled back transactions).
- Inserting a row with a given value that is higher than the current value will
  set the current value.
- The value used by the master in a statement is sent in the replication stream,
  so slaves will have the same value when re-playing the stream.
- There is no strict guarantee about ordering: two concurrent statements may
  have their commit time in one order, but their auto-incrementing ID in the
  opposite order (as the value for the ID is reserved when the statement is
  issued, not when the transaction is committed).
- MySQL has multiple options for auto-increment, like only using every N number
  (for multi-master configurations), or performance related features (locking
  that table’s current ID may have concurrency implications).
- When inserting a row in a table with an auto-increment column, if the value
  for the auto-increment row is not set, the value for the column is returned to
  the client alongside the statement result.

## Vitess Sequences

An early design was to use a single unsharded database and a table with an
auto-increment value to generate new values. However, this has serious
limitations, in particular throughtput, and storing one entry for each value in
that table, for no reason.

So we decided instead to base sequences on a MySQL table, and use a single value
in that table to describe which values the sequence should have next. To
increase performance, we also support block allocation of IDs: each update to
the MySQL table is only done every N IDs (N being configurable), and in between
only memory structures in vttablet are updated, making the QPS only limited by
RPC latency.

In a sharded keyspace, a Sequence's data is only present in one shard (but its
schema is in all the shards). We configure which shard has the data by using a
keyspace\_id for the sequence, and route all sequence traffic to the shard that
hold that keyspace\_id. That way we are completely compatible with any horizontal
resharding.

The final goal is to have Sequences supported with SQL statements, like:

```
/* DDL support */
CREATE SEQUENCE my_sequence;

SELECT NEXT VALUE FROM my_sequence;

ALTER SEQUENCE my_sequence ...;

DROP SEQUENCE my_sequence;

SHOW CREATE SEQUENCE my_sequence;
```

In the current implementation, we support the query access to Sequences, but not
the administration commands yet.

### Creating a Sequence

*Note*: The names in this section are extracted from the examples/demo sample
application.

To create a Sequence, a backing table must first be created. The columns for
that table have to be respected.

This is an example:

```
create table user_seq(id int, next_id bigint, cache bigint, primary key(id)) comment 'vitess_sequence';
```

Then, the Sequence has to be define in the VSchema for that keyspace:

```
{
  "sharded": false,
  "tables": {
    "user_seq": {
      "type": "sequence"
    },
    ...
  }
}
```

And the table it is going to be using it can also reference the Sequence in its VSchema:

```
{
  ...
  "tables" : {
    "user": {
      "column_vindexes": [
           ...
      ],
      "auto_increment": {
        "column": "user_id",
        "sequence": "user_seq"
      }
    },
```

After this done (and the Schema has been reloaded on master tablet, and the
VSchema has been pushed), the sequence can be used.

### Accessing a Sequence

If a Sequence is used to fill in a column for a table, nothing further needs to
be done. Just sending no value for the column will make vtgate insert the next
Sequence value in its place.

It is also possible to access the Sequence directly with the following SQL constructs:

```
/* Returns the next value for the sequence */
select next value from my_sequence;

/* Returns the next value for the sequence, and also reserve 4 values after that. */
select next 5 values from my_sequence;
```

## TO-DO List

### DDL Support

We want to add DDL support for sequences, as previously mentioned:

```
CREATE SEQUENCE my_sequence;

ALTER SEQUENCE my_sequence ...;

DROP SEQUENCE my_sequence;

SHOW CREATE SEQUENCE my_sequence;
```

But for now, the Sequence backing table has to be created and managed using the
usual schema management features, with the right column definitions and table comment.

Contact: vitess@googlegroups.com  **·**  
Announcements  **·**  
© 2017 Vitess powered by Google Inc
